# Interactions between Bitter Taste, Diet and Dysbiosis: Consequences for Appetite and Obesity

**DOI:** 10.3390/nu10101336

**Published:** 2018-09-20

**Authors:** Alexandria Turner, Martin Veysey, Simon Keely, Christopher Scarlett, Mark Lucock, Emma L. Beckett

**Affiliations:** 1 School of Medicine and Public Health, University of Newcastle, Ourimbah 2258, Australia; alexandria.turner@uon.edu.au (A.T.); martin.veysey@hyms.ac.uk (M.V.); 2 Hull York Medical School, University of York, Heslington YO150DD, UK; 3 School of Biomedical Sciences and Pharmacy, University of Newcastle, Callaghan 2308, Australia; simon.keely@newcastle.edu.au; 4 Hunter Medical Research Institute, New Lambton Heights 2305, Australia; 5 School of Environmental and Life Sciences, University of Newcastle, Ourimbah 2258, Australia; c.scarlett@newcastle.edu.au (C.S.); mark.lucock@newcastle.edu.au (M.L.)

**Keywords:** T2R, *TAS2R*, bitter, dysbiosis, microbiota, obesity, microbiome, diet

## Abstract

The type 2 family of taste receptors (T2Rs) detect and respond to bitter tastants. These receptors are expressed throughout the gastrointestinal (GI) tract, with location dependant roles. In the oral cavity, T2Rs are involved in the conscious perception of bitter tastants, while in the lower GI tract they have roles in chemoreception and regulation of GI function. Through these diverse roles, these receptors may be involved in modulating appetite and diet, with consequences for weight regulation and obesity. Interestingly, the concentration of T2Rs in the GI tract is greatest in the large intestine, the organ with the densest colonisation of bacteria. The gut microbiome has been the subject of intense research, as a plethora of roles linking microbiota to human health continue to be uncovered. Of particular interest is the microbial signature associated with obesity. Obesity is a leading health concern, and advances in our understanding of this disease are needed. Diet is a known modifiable factor in the development of obesity. However, diet only partially explains disease risk. Changes in microbial energy harvesting by the microbiota plays a role in obesity, and the composition of these energy harvesting populations may be controlled by taste receptors. This review explores T2Rs as a potential link between obesity and the human GI microbiome.

## 1. Introduction

Taste is one of the five fundamental senses. It is important in determining the composition of food, in terms of both identifying nutrients, and potentially harmful substances. Salts and acids are recognised by salty and sour ion channel receptors, respectively. Carbohydrates, sweeteners and protein sources are detected by the type 1 family of taste receptors (T1Rs) [1,2]. Bitter taste, which detects a variety of compounds including potential toxins, is mediated by the type 2 family of taste receptors (T2Rs) [3,4]. Humans are known to express 25 different T2Rs, which in combination are capable of detecting thousands of bitter molecules, as each T2R can bind to a range of ligands. Some of these receptors are broadly tuned, others have a narrow range, while others are yet to have their ligands identified [4]. T1Rs and T2Rs, are G-protein coupled receptors (GPCRs). The genes for each of these T2Rs are potentially polymorphic, resulting in a variety of phenotypes. This genetic variance, coupled with their key roles in health and disease, make T2Rs an important subject of investigation, which may lead to novel therapeutics.

Bitter receptors play an overall protective role in the body by triggering an adverse reaction to potentially harmful toxins and spoilt foods. However, bitter receptors may also be involved in modulating dietary intake of other foods. Healthful foods, including many vegetables, contain bitter compounds, such as isothiocyanates. As such, a genetic sensitivity to bitterness may lead to a decrease in the consumption of healthy, bitter foods. In turn, this may result in increased carbohydrate intake and weight gain [5,6]. Conversely, bitter taste acuity is often regarded as a marker for general taste acuity, and so those with a genetic sensitivity to bitter may also be more sensitive to other tastes, leading to reduced calorie intake [7,8]. Additionally, T2Rs have been shown to play important regulatory roles in the gastrointestinal (GI) tract, over and above conscious perception of bitter tastants. They are involved in glucose homeostasis, gut motility, nutrient sensing, and secretion of appetite and satiety hormones [9,10,11,12,13,14]. As such, T2Rs have crucial and complex roles roles in the body’s digestive and metabolic processes, which may alter risk for metabolic conditions including risk for diabetes and obesity [15]. 

A relationship has been established between certain microbial signatures in the GI tract and risk for obesity [16]. A decrease in the relative proportions of *Bacteroidetes* and a subsequent increase of *Firmicutes* in the colon is associated with increased risk for obesity [17,18]. A number of mechanisms may explain these associations, including modulations of energy uptake. In mice, an increased harvesting of energy from the diet has been demonstrated in genetically obese mice, compared to lean mice [19]. Germ-free mice are also resistant to diet-induced obesity, compared to mice with a normal gut microbiota, in a fasting-induced adipose factor dependant manner. This persistently lean phenotype was associated with increased fatty acid metabolism [20]. These data support the involvement of the GI microbiota in the regulation of energy uptake.

In addition to its roles in the regulation of energy harvesting and metabolism, the GI microbiota may also contribute to risk for obesity via modulation of behaviour. Observational data suggest that gut bacteria may also play a role in the determination of dietary preferences via modulation of taste receptor expression [14,21]. For example, increased colonic expression of T1Rs has been demonstrated in germ-free mice, leading to an increased intake of a sweet solution [22].

Taste, dietary choices and GI microbiota are often analysed as individual contributing factors in the aetiology of obesity. However, obesity is a complex and multifactorial condition, and elucidating the mechanisms at play requires considerations not only of the additive impacts of these factors, but also the interactions between them. Furthermore, the potential role for extra-oral T2Rs in the aetiology of obesity is easily overlooked, as they are not involved in the detection of energy-containing nutrients. Therefore, these elements are reviewed together here, with a view to creating a more complete picture of obesity in regard to bitter taste genetics, diet, and microbial compositions. These factors may interact in a multidirectional manner.

## 2. Bitter Taste and Obesity 

**Figure 1:**
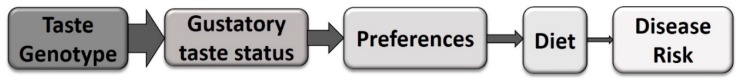
The standard paradigm linking taste genetics to risk for disease. Genotype is a major determinant of gustatory taste phenotype, which in turn determines taste preferences. This impacts dietary choices, thus impacting on the risk for diseases with dietary risk factors.

Polymorphisms in T2R genes are associated with modulation of risk for a range of diseases. These associations can partly be attributed to the modulation of food preference, as genetic variance in taste receptors are a determinant of dietary preferences, and as such play a role in determining dietary habits (Figure 1). However, this standard paradigm does not completely explain the observed relationships between taste genetics and disease risks.

Haplotypes of *TAS2R38*, or phenotypes for sensitivity to the receptors ligand 6-n-propylthiouracil (PROP), are often used to define taster status. Multiple non-synonymous single nucleotide polymorphisms have been identified in the *TAS2R38* gene [23]. However, three of these (rs713598, rs1726866 and rs10246939), resulting in three amino acid substitutions (A49P, A262 V and V2961), have been identified as being primarily responsible for the observed phenotype. Two haplotypes, Ala-Val-Ile (AVI) and Pro-Ala-Val (PAV), are common. Individuals with at one or more copies of the PAV haplotype are more sensitive PROP and are more likely to be classified as tasters or supertasters in phenotypic testing. Super-tasters and tasters have strong and slight aversions to PROP, respectively. 

Conversely, those who are homozygous for the AVI haplotype are more likely to be unable to detect PROP and are classified as non-tasters. Approximately 30% of people are classified as non-tasters in this way [24,25]. In addition to being less sensitive to bitter tastes, non-tasters are less sensitive sweet tastes, the astringency of alcohol, the pungency of chillies and the texture of fats [25,26]. Therefore, PROP sensitivity is often regarded as a general marker of taste acuity [27].

Taste acuity and innate preferences are determinants of dietary intakes. As such, it has been hypothesised that super-tasters may be more prone to obesity, due to a reduced intake of healthy, bitter tasting, antioxidant-rich foods [28]. However, it appears that super-tasters may also have a corresponding lower intake of high fat and high sugar foods [5]. 

Several studies have attempted to relate taster status to weight status. However, results are mixed, and a clear link has not been established [29,30]. Several studies have shown significant relationships between taster status and body mass index (BMI), with higher BMIs found in non-tasters [5,8,31,32]. However, relationships may exist in some sub-populations and not others, as significant relationships have only been demonstrated in females [8,31,32], and in non-obese individuals [5,8,32].

A large observational study reported a significant association between risk for obesity and the *TAS2R38* AVI/AVI haplotype in females [6]. This was also associated with increased eating disinhibition in non-tasters [6]. These associations were not observed in male participants. The relationship between taster status and adiposity is further supported by a study that found a significant association between three *TAS2R38* variants and body fat percentage in females only [33]. The sex-specificity of these observations may be explained by an interaction between sex hormones and taste signalling, with consequences for appetite and intake. Overall, high taste acuity may reduce dietary overconsumption and therefore reduce the risk for obesity, with a stronger association in females [34]. While *TAS2R38* has been well studied, more information is needed on the relationship between other T2R gene polymorphisms and risk for obesity. Polymorphisms in other T2R genes have been linked to other conditions, including cardiovascular disease, altered thyroid function and longevity [35,36,37], and these may also be linked to weight and metabolic health.

**Figure 2:**
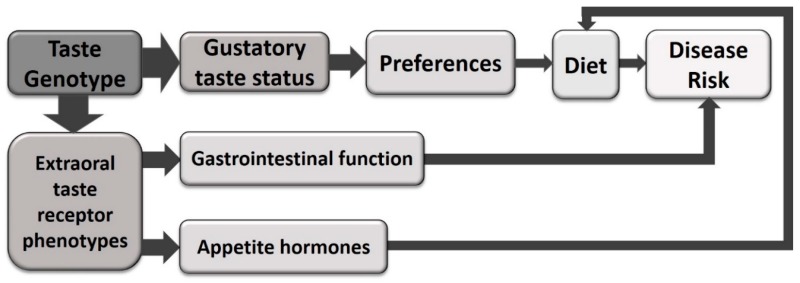
The relationships between taste and disease risk may not be fully explained via the standard paradigm of modulation of taste preferences and diet. Evidence is also emerging linking taste genetics to disease risk via extraoral functions, such as modulation of gastrointestinal function (including motility and fluid secretion) and regulation of appetite hormones.

While modulation of dietary risk factors may explain some of the links between taster status and risk for disease, non-gustatory functions of taste receptors may also be involved. These functions include modulation of gastrointestinal functions, such as fluid secretion and gut motility, and regulation of appetite signalling (Figure 2). It may be intuitive that T1Rs (detecting energy-containing carbohydrates and amino acids) play a role in the development of obesity. However, it is becoming apparent that T2Rs are also involved in glucose regulation and the release of appetite hormones [14,34], including ghrelin (hunger-inducing), cholecystokinin (CCK; involved in satiation and gut motility) and glucagon-like peptide-1 (GLP-1; involved in glucose regulation) [12,15,33].

These mechanisms have been demonstrated in cell culture and murine models. Intragastric administration of bitter ligands, which by-passes the gustatory taste receptors, results in increased food intake and increased ghrelin secretion. However, this appetite modulatory effect is reduced in ghrelin-receptor knockout mice, compared to controls [12]. In enteroendocrine cell models, stimulation with bitter ligands increased the secretion of CCK and induced GLP-1 secretion [14,38]. 

**Figure 3:**
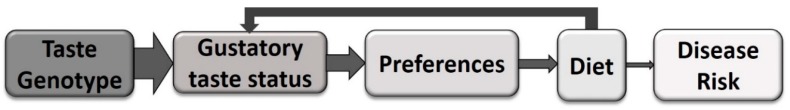
The relationship between taste status and diet is bi-directional. Dietary stimuli can also modulate the expression of taste receptors, modulating taster status which feeds back to modulate preferences and intake.

Furthermore, a role for T2Rs in glucose and insulin regulation has been demonstrated in human cohorts. Several T2R polymorphisms have been determined to be related to increased risk for diabetes and resulted in a disruption of glucose homeostasis [14]. In an Amish Family Diabetes study, a *TAS2R9* polymorphism was associated with altered GLP-1 secretion and glucose regulation. In a subsequent study, the *TAS2R38* AVI/AVI haplotype was similarly associated with high plasma glucose levels [33]. Due to a key role in the regulation of appetite, glucose metabolism and gut motility, genetic variations in T2Rs may contribute both to the development of obesity and risk for diabetes. In addition to modulation of function via genetic variance, expression of T2Rs can also be modulated in response to stimulation. In human studies, *TAS2R38* has been shown to be overexpressed in the colon of overweight and obese individuals when compared to those of normal weight [39]. Furthermore, a murine study revealed that the expression of *tas2r138* (equivalent to human *TAS2R38*) was increased in the colon after a long-term high-fat diet [40]. Therefore, it is reasonable to conclude that a bidirectional interaction exists, with diet influencing the expression of taste receptors in the GI tract, as well as taste genetics modulating dietary preferences (Figure 3). It is likely that multiple T2Rs are involved in these associations, with genetic variance and expression of multiple receptors interacting to modulate risk. However, to date, research has focused only on the well characterised TAS2R38 receptor. Additional studies are needed including assessment of multiple receptors and multiple polymorphisms to full elucidate these relationships and identify potential therapeutic targets.

While there is evidence to support the standard paradigm that taste genetics influence weight and appetite via modulation of food preference, there is now also emerging evidence that dietary compounds may stimulate taste receptors throughout the GI tract. This has effects on taste receptor expression and GI function, with potential consequences for the GI microbiota, including known interactions between microbiota and GI functions such as motility [41]. 

## 3. Obesity and the Gut Microbiome

The prevalence of obesity continues to increase worldwide and is linked to increased incidences of diabetes, metabolic syndrome, certain cancers, reduced quality of life and increased socioeconomic burdens [42]. It is known that diet is strongly linked to risk for obesity, and that diet also modulates the GI microbiome. This relationship was established over 100 years ago [43] and has been continually reinforced [44,45]. More recently, it has been suggested that gut bacteria may be involved in the regulation of host appetite. Bacterial components and metabolites have been shown to stimulate satiety [46], which suggest a role for the microbiome in appetite control. 

Manipulation of the GI microbiota affects adiposity and metabolism [21]. Over a decade ago, it was demonstrated that germ-free mice were protected against diet-induced obesity in contrast to wild-type mice with a wild-type gut microbiome [20]. It was shown that, when the microbiota from obese mice is transferred into germ-free mice, a significant increase in body fat was observed, compared to germ-free mice colonised with a “lean” microbiota [19]. This demonstrates that the obese microbiome has an increased capacity to harvest energy from the diet [19,20]. From these studies, a clear link between the composition of the gut microbiome and obesity has been established. 

An imbalance in the ratio of *Bacteroidetes* to *Firmicutes* phyla is more often observed in obese subjects than controls. Genetically obese mice displayed a 50% reduction in *Bacteroidetes* and a proportional increase in *Firmicutes*, compared to lean mice and regardless of kinship, after all being fed the same polysaccharide-rich diet [17]. This idea is supported by additional data showing a relative increase of *Bacteroidetes* in obese individuals, and a proportional increase with weight loss [18]. Furthermore, a high faecal concentration of *Staphylococcus* species was associated with high energy intake in children [47]. Overall, despite individual variation, obesity displays a specific microbial signature. 

There is a demonstrated link between diet-induced obesity and a bloom of *Mollicutes* (a class of *Firmicutes*). These findings suggest that dysbiosis is not only caused by but can, in fact, precede obesity. It was demonstrated via mouse models that a *Mollicute* bloom occurred in response to diet-induced obesity and that, when this microbiome was transferred into lean germ-free mice, increased adiposity was observed [16]. Overall, an increase in the number of *Firmicutes*, and a reduction in *Bacteroidetes* is representative of the obese microbiome, and dysbiosis is thought occur prior to weight gain and not only in response weight-related changes [16,19]. 

There may be a number of mechanistic pathways involved in the association between GI microbiota and weight. GI bacteria produce short-chain fatty acids (SCFAs) as a metabolic product, and these have been implicated as aetiological factors in obesity. SCFAs are thought to stop the accumulation of fat in adipose tissue, so a decreased level is believed to contribute to obesity [48]. A previous study showed that animals supplemented with butyrate while on a high-fat diet displayed reduced insulin resistance and obesity [49]. Therefore, SCFAs may offer some protection against metabolic conditions. There is a high likelihood that T2Rs and other taste receptors form part of these mechanistic pathways.

## 4. T2RS as A Link between the Microbiome and Obesity 

The GI functions that are regulated by T2Rs, including GI motility, appetite regulation, nutrient uptake and fluid secretion, also have a major effect on the gut microbiota. Additionally, dietary modulation does not fully explain the relationships between taste status and risk for disease. Given the diverse roles of T2Rs in the GI tract, and that there a high concentration of T2Rs in the colon, it has been speculated that these receptors may respond to alterations of the gut microbiota, which may have consequences for disease risk.

An interaction between T2Rs and bacteria has been demonstrated in another organ system. In the lungs, T2Rs play a protective role against bacterial invasion. They detect bacterial signalling molecules and initiate immune responses. T2Rs interact with and respond to noxious substances, bacteria and bacterial quorum-sensing molecules in the respiratory tract [50,51,52,53]. Detection of upper respiratory tract irritants by trigeminal nerve endings induces protective responses. However, the epithelium also contains solitary chemosensory cells that express T2Rs and their downstream signalling molecules. It was shown that these cells respond to both the bitter ligand denatonium, and to bacterial signals by increasing intracellular Ca^2+^. Furthermore, genetic knockout of Gα-gustducin or TrpM5 (Transient receptor potential cation channel subfamily M member 5, essential elements of the T2R signalling cascade) eliminates the trigeminal response [51]. Overall, T2Rs are involved in the regulation of the mucosal innate immune system [53]. Due to the nature of this interaction with potential pathogens, it is hypothesised that TASRs in the GI tract may also interact with GI microbiota.

Bacteria are thought to be able to modulate dietary preferences. GI microbiota may alter expression of taste receptors, and therefore influence food preferences [14,21,54]. A study demonstrated that T1Rs were overexpressed in the intestinal epithelium of germ-free mice and that these mice preferred a higher concentration sucrose solution compared to WT mice [22]. This suggests a role of extra-oral taste receptors in food preference, which may be influenced by gut bacteria. 

The ability of GI microbiota to influence eating behaviour has been previously reviewed [54]. This study suggested that microbes may be able to alter host eating patterns, potentially via the manipulation of taste receptors. Furthermore, gastric bypass surgery, undertaken as a treatment for obesity, is known to modulate the GI microbiota [55]. This procedure is also known to alter taste receptor expression levels [54,55]. This supports the existence of an interaction between the GI microbiota, and taste receptor expression. Such interactions may occur via bitter bacterial metabolites (SCFAs) and signalling molecules (quorum-sensing molecules) acting as T2R ligands. SCFAs interact with other GPCRs in the body [56], and signalling molecules have been shown to activate T2Rs in the respiratory tract [53]. 

The human microbiota is known to vary with aging [57,58,59], and it has been proposed that these changes are associated with risk for obesity through the lifespan [60]. Many reasons for microbiota to vary with age have already been proposed, including changes in diet linked with life-stages. However, this may also be linked to magnitude of tastant detection as taste perception is also known to change with age. Tasting is more acute in children than adults, and perception declines in old age [61,62]. This may occur as a response to, or be a causative factor in changing microbial profiles. However, the direct links between taste, aging, obesity and the gastrointestinal microbiota are yet to be investigated.

Some artificial sweeteners, such as saccharin and acesulfame potassium, stimulate T2Rs as well as T1Rs [63]. Interestingly, artificial sweetener use is also associated with changes in host microbiota profiles, decreased satiety signalling, altered glucose homeostasis, increased calorie intake and weight gain [64]. However, the direct roles of extraoral T2Rs modulating these changes in response to artificial sweetener use are yet to be investigated. 

**Figure 4:**
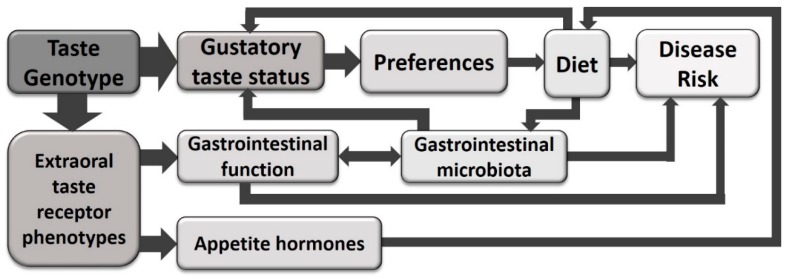
The potential interactions that link taste, diet, the gastrointestinal microbiota and disease risk are multifaceted and complex, with numerous bidirectional associations.

Overall, a role for T2Rs in the complex relationship between gut microbiota and obesity may exist (Figure 4). However, further research is needed to establish this. It is important that future research assesses the expression and genotypes of multiple T2R in the same subjects, as it is likely that multiple receptors act in concert to modulate function and therefore disease risk. 

As research methods and technology for assessing genetic variance [65,66] and microbial profiles [67,68] become more advanced, it becomes more feasible to investigate the interactions between taste genetics, taste receptor expression, GI microbiota, diet and risk for obesity and other diseases. It is important as we move forward to move away from the reductionist approach of studying single genes, nutrients or microbial families and to develop a more integrated approach to studying these interactions. Animal models will be vital to the advancement of this field, as collecting samples from the GI tract is invasive in humans. Advanced statistical modelling is needed to assess interactions as well as additive effects of these factors in determining disease risk. 

## 5. Conclusions 

The human body is sustained by a series of finely-tuned and balanced interactions. The interactions among diet, taste and the gut microbiome are multi-directional, and each interaction may modify disease risk. T2Rs are involved in an array of mechanisms that can lead to obesity. We propose that extra-oral bitter receptors interact with gut bacteria to influence food intake and gut motility, both of which influence the risk for the development of obesity. The details of such an interaction remain to be elucidated. 

Overall, the evidence suggests that T2Rs play a role in maintaining a balance among diet, weight and a healthy microbiome. If this role can be defined, T2Rs could be manipulated to prevent or treat obesity. GPCRs are already a major drug target [69]. Consequently, the development of drugs to upregulate or block T2Rs circumstantially has therapeutic potential. Therefore, further investigations into the role of T2Rs in the relationship between obesity and the gut microbiome are essential.
